# Majorana gets an iron twist

**DOI:** 10.1093/nsr/nwy143

**Published:** 2018-11-22

**Authors:** Lingyuan Kong, Hong Ding

**Affiliations:** Institute of Physics, Chinese Academy of Sciences, China

A magical coincidence of condensed-matter physics occurred in 2008. In February of that year, a new kind of high-*T*_c_ (26 K) superconductivity was reported in the fluorine-doped iron-based superconductor (FeSC) La[O_1-x_F_x_]FeAs [1], starting the ‘Iron Age’ of superconductivity. In the following April, a 3D topological insulator (TI) was verified in Bi–Sb alloy [2], triggering a worldwide wave of searching for more 3D TI materials. A little earlier, in that March, a new hope was also raised in the Majorana community. An ingenious idea was proposed by Fu and Kane [3] for creating self-conjugate Majorana bound states (MBSs), a condensed-matter version of the famous Majorana fermion, in a TI/s-wave superconductor heterostructure, which is much easier to realize in experiments than the previously proposed p-wave superconductor platform. These topics soon became three major rivers in condensed-matter physics, and they come together with the recent discovery of topological band structure and Majorana bound states in a simple FeSC.

This new development was partially motivated by the perspective of fault-tolerant topological quantum computing based on non-Abelian statistics of MBSs. Several material platforms were proposed to harbor MBSs in the early days, such as intrinsic p-wave superconductors, and multiple heterostructures combining strong spin–orbit coupling (SOC) and superconductivity. The rare nature and heterostructure of those platforms caused difficult conditions for observing and manipulating MBSs, such as complicated device structure, low *T*_c_, and strong quasiparticle poisoning largely due to the small ratio between the superconducting gap (*Δ*) and the Fermi energy (*E*_F_). It would be a wonder to combine the material simplicity, high *T*_c_, and large *Δ*/*E*_F_ ratio into one platform. Recently, two papers published in *Science*, reporting observations of superconducting topological surface states [4] and MBSs [5] in FeTe_0.55_Se_0.45_ (*T*_c_ ∼ 15 K), finally realized this wonder.

In 2014, the novel intertwining between non-trivial band topology and high-*T*_c_ superconductivity was first proposed, although not verified experimentally, on stressed monolayer FeSe [6]. The topological insulator phase emerges around the zone corner in which a topological bulk gap is induced by a combination of the effects of SOC and substrate stress. In 2015, angle-resolved photoemission spectroscopy (ARPES) evidence of p–d orbital inversion [7] and scanning tunneling spectroscopy (STS) evidence of iron impurity zero-bias conductance peaks (ZBCPs) were found on bulk crystal FeTe_0.55_Se_0.45_ [8], propelling to the fore the idea of achievable topological insulating band structure by chemical substitution in a bulk FeSC material. The definitive evidence came from high-resolution spin-resolved ARPES in 2018 that observed a spin–momentum locking pattern of the Dirac surface band, a hallmark of a topological insulator, in FeTe_0.55_Se_0.45_ [4]. More remarkably, this Dirac surface band opens a reasonably sized superconducting gap below the bulk *T*_c_, which is identical to the interfacial state in the Fu–Kane model [3], but with a clear advantage that *Δ*/*E*_F_ in FeTe_0.55_Se_0.45_ is much larger than the one in an ordinary Fu–Kane device.

**Figure 1.**
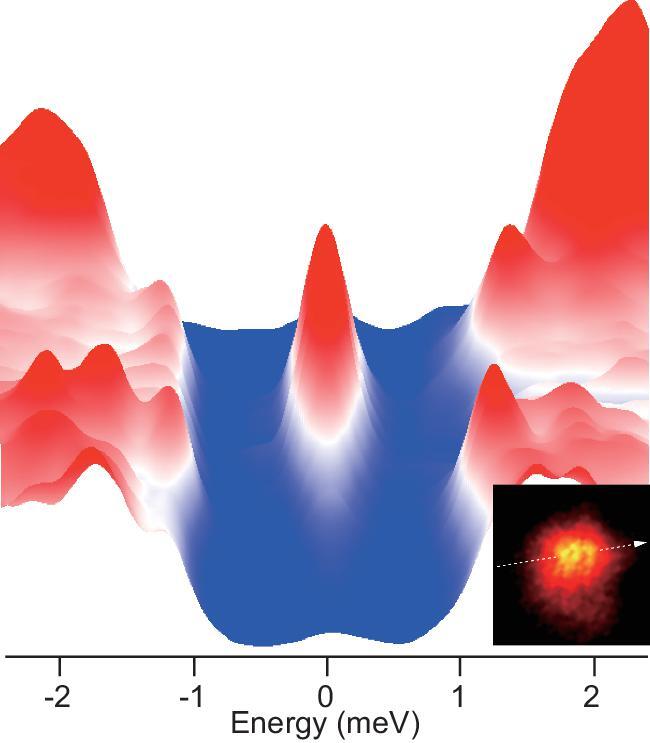
A robust Majorana bound state observed in FeSC. A 3D display of a line-cut intensity plot of Majorana bound states on the vortex core (inset) of FeTe_0.55_Se_0.45_. It is visualized.

This advantage, which would separate the non-topological bound states from the MBS in a superconducting vortex core, immediately motivated a high-resolution STM study on FeTe_0.55_Se_0.45_ [5]. Indeed, a spatial non-split ZBCP was observed in the vortex cores of FeTe_0.55_Se_0.45_ [5], which is a fingerprint of the MBS (Fig. 1). The behaviors of the observed ZBCPs were studied carefully in FeTe_0.55_Se_0.45_ [5] under different temperatures, magnetic fields and tunneling barriers. Its full width at half maximum approaches the STM resolution limit, indicating a single peak. The intensity line profile of the ZBCPs was reproduced very well by a theoretical Majorana wave function with the parameters extracted from the experimental values of the Dirac surface state. An additional suppression mechanism of the ZBCPs is also identified by temperature-dependent measurements. Although an unequivocal demonstration of the Majorana behavior needs more evidence, those results drew a self-consistent picture in which the observed ZBCPs are the MBSs associated with the surface Dirac electrons.

The unique features of the FeSC platform for Majorana research have attracted much attention from the community. There are many important unsolved issues remaining for future studies, such as MBS-induced spin-selective Andreev reflection, quantized conductance of 2*e*^2^/*h*, the Majorana interference pattern, and non-Abelian statistics demonstrated in a Majorana braiding process. Another puzzle that needs to be resolved is the absence of MBS on many vortices in FeTe_0.55_Se_0.45_ [5]. Although the MBSs in the vortex of Fe(Te, Se) are purer and the large quasiparticle gap may protect the fermion parity information carried by a pair of MBSs robust against perturbations, which is a good feature required by fault-tolerant quantum computing, a realistic method for experimentally realizing braiding for MBSs in vortices has yet to be proposed.

The scenario of MBSs in a vortex was recently put forward to explain the robust ZBCPs observed on iron-impurity sites of FeTe_0.55_Se_0.45_, suggesting that an anomalous vortex core is induced by a single impurity without an extrinsic magnetic field [9]. The successes with FeTe_0.55_Se_0.45_ have also prompted researchers to pursue better platforms with higher *T*_c_, such as Fe(Te, Se) monolayer [10], Li(Fe, Co)As [11], and (Li, Fe)OHFeSe [12], further strengthening the miraculous marriage between topology and high-*T*_c_ superconductivity in FeSCs.
